# Methylmercury induces the expression of TNF-α selectively in the brain of mice

**DOI:** 10.1038/srep38294

**Published:** 2016-12-02

**Authors:** Miyuki Iwai-Shimada, Tsutomu Takahashi, Min-Seok Kim, Masatake Fujimura, Hitoyasu Ito, Takashi Toyama, Akira Naganuma, Gi-Wook Hwang

**Affiliations:** 1 Laboratory of Molecular and Biochemical Toxicology, Graduate School of Pharmaceutical Sciences, Tohoku University, Sendai 980-8578, Japan; 2 Center for Health and Environmental Risk Research, National Institute for Environmental Studies, Onogawa16-2, Tsukuba, Ibaraki 305-8506, Japan; 3 School of Pharmacy, Tokyo University of Pharmacy and Life Sciences, 1432-1 Horinouchi, Hachioji, Tokyo 192-0232, Japan; 4 Department of Inhalation Toxicology Research, Korea Institute of Toxicology, Jeonbuk 56212, Republic of Korea; 5 Department of Basic Medical Science, National Institute for Minamata Disease, Kumamoto 867-0008, Japan; 6 Department of Informative Clinical Medicine, Gifu University Graduate School of Medicine, 1-1 Yanagido, Gifu 501-1194, Japan

## Abstract

Methylmercury selectively damages the central nervous system (CNS). The tumor necrosis factor (TNF) superfamily includes representative cytokines that participate in the inflammatory response as well as cell survival, and apoptosis. In this study, we found that administration of methylmercury selectively induced TNF-α expression in the brain of mice. Although the accumulated mercury concentration in the liver and kidneys was greater than in the brain, TNF-α expression was induced to a greater extent in brain. Thus, it is possible that there may exist a selective mechanism by which methylmercury induces TNF-α expression in the brain. We also found that TNF-α expression was induced by methylmercury in C17.2 cells (mouse neural stem cells) and NF-κB may participate as a transcription factor in that induction. Further, we showed that the addition of TNF-α antagonist (WP9QY) reduced the toxicity of methylmercury to C17.2 cells. In contrast, the addition of recombinant TNF-α to the culture medium decreased the cell viability. We suggest that TNF-α may play a part in the selective damage of the CNS by methylmercury. Furthermore, our results indicate that the higher TNF-α expression induced by methylmercury maybe the cause of cell death, as TNF-α binds to its receptor after being released extracellularly.

Methylmercury is an environmental pollutant known as the cause of Minamata disease, and severe central nervous system damage^1^,^2^,^3^. In sea- and fresh water, methylmercury bioaccumulates through the food chain to relatively high concentrations in the muscle of fish. As such, there are concerns that excessive consumption of fish and, hence, methylmercury by expectant mothers may affect the brain development of the fetus^4^,^5^,^6^,^7^. Nevertheless, despite global concerns over the health effects of methylmercury, the mechanism involved in its toxicity is not understood.

Microglia and astrocytes, types of brain cells, regulate the neuroprotective immune response that participates in maintaining brain homeostasis. However, there are cases in which they have instead been shown to be the cause of damage to neurons. In recent years, glial contributions have been noted in the progression and exacerbation of neurodegenerative disorders including Alzheimer’s disease, amyotrophic lateral sclerosis, and Parkinson’s disease^8^,^9^,^10^,^11^.

Activated microglia and astrocytes produce inflammation mediators (e.g., prostaglandin, nitric oxide), inflammatory cytokines and other products. The production of inflammatory cytokines is known to damage neurons through the invasion of inflammatory cells and cytotoxic T cells^12^,^13^. Cytokines play an important role in intercellular signaling, and over 100 types have been described, including tumor necrosis factor (TNF), transforming growth factor (TGF), interleukins, interferons, and chemokines^14^,^15^. Recently, Rostene *et al*.^16^ reported the relationship between CCL2, a chemokine, and methylmercury toxicity. Rostene *et al*. investigated the effect of adding recombinant CCL2 to culture media on the methylmercury sensitivity of primary rat neurons. Instead of CCL2 reducing methylmercury toxicity, they found it amplified toxicity at high concentrations^16^. We have previously demonstrated that expression of CCL3 and CCL4, which belong to the inflammatory chemokine family, is selectively induced in the brains of mice as a result of methylmercury administration^17^. Further, Koizumi *et al*. reported that methylmercury induces IL-6 expression in astrocytes, and that IL-6 exhibits a defensive effect against the neuronal damage caused by methylmercury^18^. Taken together, these results suggest that cytokines produced in the brain are involved in the neurotoxicity of methylmercury; however, there are many unresolved questions regarding the role of cytokines in the damage caused to the central nervous system by methylmercury.

The TNF superfamily (TNFSF) are representative inflammatory cytokines that participate in processes such as cell survival, induction of apoptosis, the inflammatory response, and cell differentiation. Approximately 20 molecular species have been identified, including cytokines that participate in the induction of apoptosis, such as: TNF-α, lymphotoxin α, the Fas ligand (FasL) and TNF-related apoptosis-inducing ligand (TRAIL), as well as the RANK ligand (RANKL) and the CD30 ligand (CD30L)^19^,^20^,^21^. Until now, there have been no detailed investigations into the relationship between methylmercury toxicity and the TNFSF. Thus, in this study we examined which members of the TNF superfamily were induced in the brains of mice treated with methylmercury.

## Results

### The effect of methylmercury on TNFSF expression in mouse tissue

**Figure 1:**
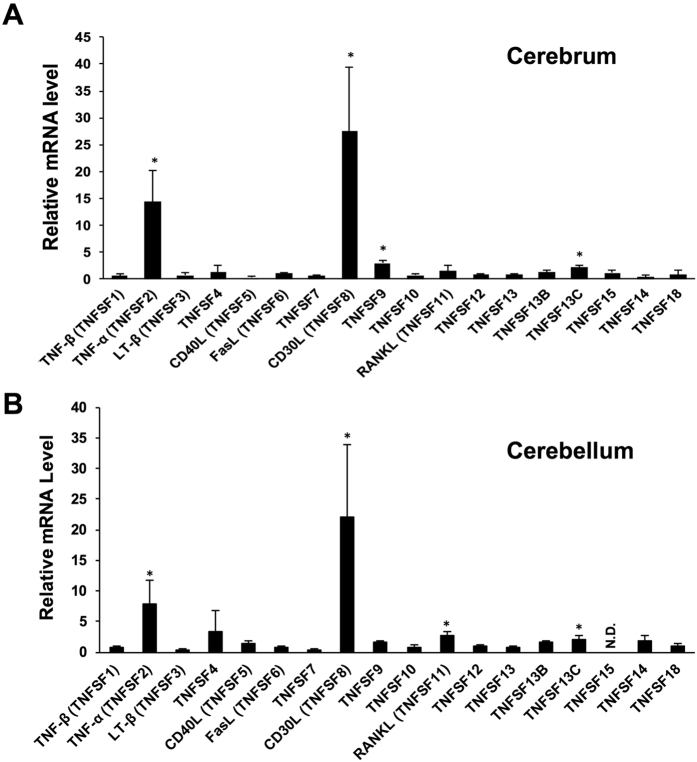
Effect of methylmercury on TNFSF mRNA levels in the brains of mice. Male mice (n = 5) were injected subcutaneously with methylmercuric chloride (25 mg/kg, one shot). Their cerebrum and cerebellum were then dissected 7 days after injection. qPCR was used to determine the level of TNFSF mRNA in the cerebrum (**A**) and in the cerebellum (**B**). Each mRNA level was normalized to that of GAPDH. Data represent fold-changes in mRNA levels (mean ± S.D.). *Indicates a significant difference (P < 0.05) compared with the control group. Data were analyzed using student’s t-tests. N.D.: not detected.

Male C57BL/6 mice were injected subcutaneously with methylmercuric chloride (25 mg/kg/day, one shot). Seven days after methylmercury administration, the cerebrum and cerebellum were dissected and any changes in the expression of 18 TNFSF genes were analyzed using quantitative PCR. The expression of TNF-α (TNFSF2) and CD30L (TNFSF8) were found to have increased markedly in both the cerebrum and cerebellum after methylmercury administration (Fig. 1). Additionally, although to a lesser extent than TNF-α and CD30L, there was a significant increase in the expression of TNFSF9 and TNFSF13C in the cerebrum (Fig. 1A), and of the RANKL (TNFSF11) and TNFSF13C in the cerebellum (Fig. 1B).

**Figure 2:**
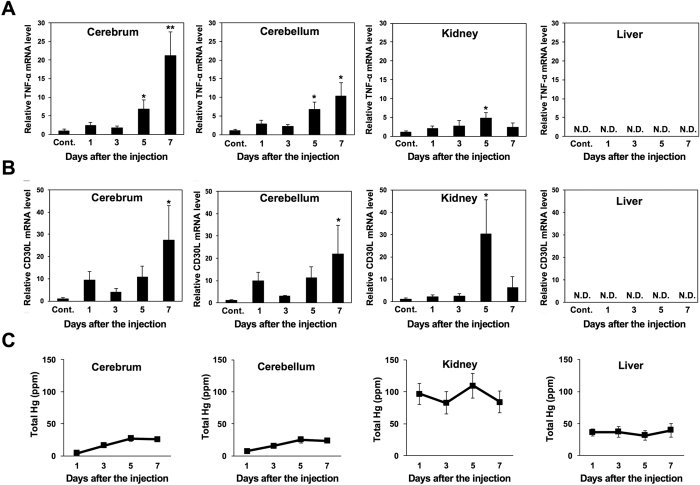
Effect of methylmercury on the levels of TNF-α and CD30L mRNA in different mouse tissues. Male mice (n = 5) were injected subcutaneously with methylmercuric chloride (25 mg/kg, one shot). Each tissue was dissected 1, 3, 5 or 7 days after the injection. qPCR was used to determine the levels of TNF-α (**A**) and CD30L (**B**). Statistical differences *P < 0.05 and **P < 0.01 compared with the control group (Cont.). Data were analyzed using student’s t-tests. N.D.: not detected. (**C**) Mercury accumulation in various organs of methylmercury-treated mice. Data represent the mean ± S.D.

Next, methylmercury was administered to mice under the same conditions as the experiment depicted in Fig. 1, and the changes in TNF-α and CD30L expression in the cerebrum, cerebellum, liver, and kidneys was measured on the first, third, fifth, and seventh days. Under this experimental protocol, a significant decrease in mouse body weight was observed on the fourth day, reaching an average weight reduction of about 13% by the seventh day (data not shown). TNF-α expression was found to increase significantly from the fifth day in the cerebrum and cerebellum, rising further on the seventh day. TNF-α expression in the kidneys also rose approximately five-fold on the fifth day, but the rate of increase was lower when compared with the increase in the cerebrum and cerebellum (Fig. 2A).

CD30L expression in the cerebrum and cerebellum was significantly elevated on the seventh day; however, by contrast to TNF-α, CD30L expression also rose on the fifth day in the kidneys at approximately the same rate as in the cerebrum and cerebellum (Fig. 2B). The expression of both TNF-α and CD30L in the liver was below the detection threshold (Fig. 2A and B). Meanwhile, the mercury concentrations in the various tissues of the mice were found to be higher in the liver and kidneys than in the cerebrum and cerebellum (Fig. 2C). The above results indicate that methylmercury selectively induces TNF-α expression in brain tissue. Consequently, it is possible that a mechanism for the selective induction of TNF-α expression by methylmercury may exist in the brain.

### NF-κB contribution to the induction of TNF-α expression by methylmercury

**Figure 3:**
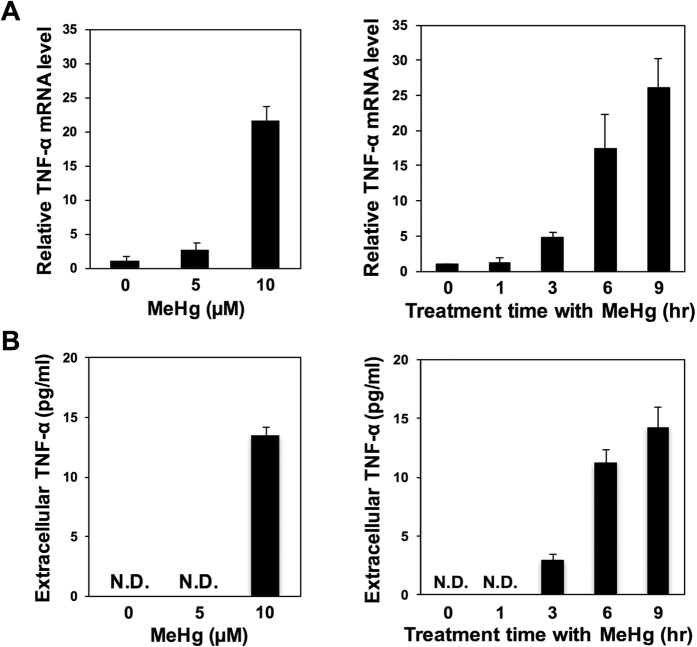
Effect of methylmercury on the expression of TNF-α in mouse neural stem cells. C17.2 cells (4 × 10^5^ cells/2 mL) were treated with the indicated concentrations methylmercuric chloride (MeHg) for 6 hr (left), and with 10 μM MeHg for the indicated times (right). (**A**) TNF-α mRNA levels were measured by qPCR. (**B**) TNF-α protein secreted into culture media was analyzed using ELISA. Data represent the mean ± S.D. of three replicates. N.D.: not detected.

**Figure 4:**
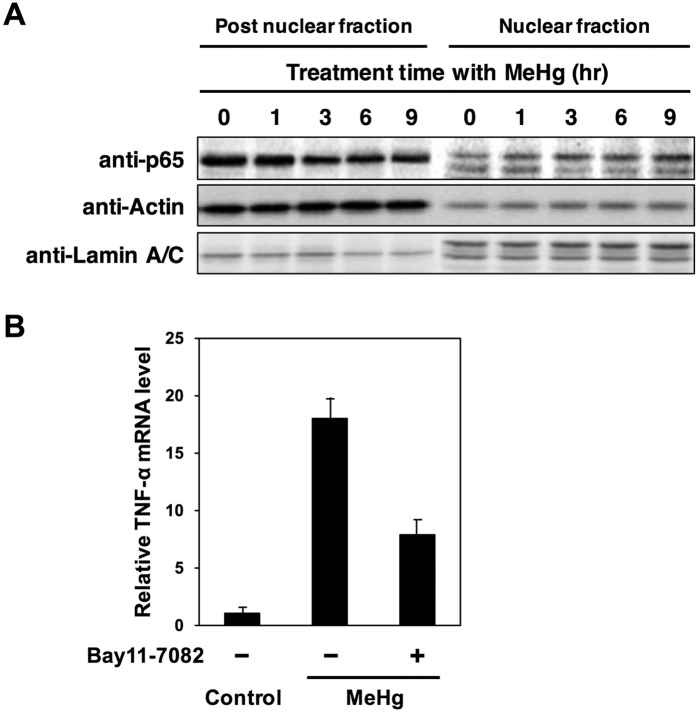
Involvement of NF-κB in the induction of TNF-α expression by methylmercury in mouse neural stem cells. (**A**) Effect of methylmercury on nuclear translocation of p65, a subunit of NF-κB, in mouse neural stem cells. C17.2 cells (4 × 10^5^ cells/2 mL) were treated with 10 μM MeHg for the indicated times. Levels of p65 in nuclear and post-nuclear fractions were monitored by immunoblotting. Lamin A/C and GAPDH were used as nuclear and post-nuclear markers, respectively. (**B**) Effect of Bay11-7082, an inhibitor of NF-κB, on the induction of TNF-α expression by methylmercury. C17.2 cells (4 × 10^5^ cells/2 mL) were pretreated, or not treated, with 5 μM Bay11-7082, which was dissolved in dimethyl sulfoxide, before the cells were exposed 2 hr later to 10 μM MeHg. TNF-α mRNA levels were measured by qPCR. Data represent the mean ± S.D. of three replicates.

We found a concentration- and time-dependent induction of TNF-α expression resulting from the treatment of C17.2 cells, which are mouse neural stem cells, with methylmercury (Fig. 3A). The TNF-α levels in the culture medium increased alongside the induction of TNF-α expression, as determined by measuring TNF-α levels in the culture medium, using the ELISA method (Fig. 3B). We also confirmed the expression of TNF receptor 1 (TNFR1) that is major a TNF-α receptor in C17.2 cells (data not shown). This indicates that C17.2 cells may have an autocrine signaling of TNF-α. NF-κB is known as a transcription factor involved the expression of a number of types of cytokines^22^,^23^. NF-κB is a complex of both p65 (RELA) and p50, and is known to migrate into the nucleus due to various stimuli, and exhibit transcription activation^24^,^25^,^26^. In this study, where we investigated the effect of methylmercury on the intracellular distribution of p65, we found that the level of p65 decreased in the post-nuclear fraction as a result of methylmercury treatment, while its concentration increased in the nuclear fraction (Fig. 4A). From this result we hypothesize that methylmercury activates NF-κB as a result of promoting nuclear translocation of NF-κB. When an NF-κB inhibitor (Bay11-7082) was added to the culture medium prior to methylmercury treatment, TNF-α expression was suppressed by approximately 50% (Fig. 4B). We also confirmed that the decrease of level of IκB, an inhibitor of NF-κB, by methylmercury was hardly observed under this condition (data not shown). From these results, we conclude that NF-κB is partially involved in the induction of TNF-α expression by methylmercury in C17.2 cells.

### Autocrine effect of TNF-α participates in expression of methylmercury toxicity in C17.2 cells

**Figure 5:**
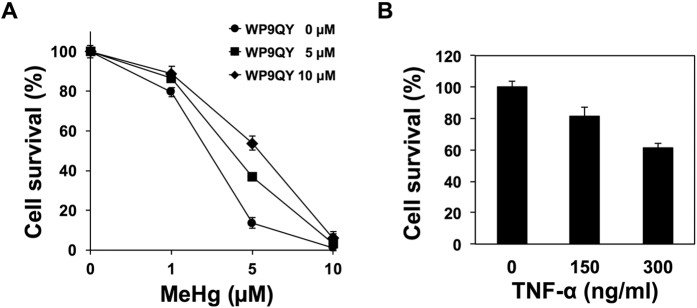
Involvement of extracellular TNF-α in the methylmercury toxicity of mouse neural stem cells. (**A**) Effect of TNF-α antagonist on the sensitivity of C17.2 cells to methylmercury. C17.2 cells (1 × 10^4^ or 5 × 10^3^ cells/well) were plated into 96-well plates, and cultured in 100 μL aliquots of the medium for 24 hr. WP9QY, a TNF-α antagonist, was then added to the cells, and 1 hr later exposed to MeHg at the indicated concentrations for 24 hr. Cell survival was determined using an Alamar blue assay. Data represent the mean ± S.D. of three replicates. (**B**) Effect of recombinant TNF-α on cell survival. C17.2 cells (3 × 10^3^ cells/well) were plated into 96-well plates, and cultured in 100 μL aliquots of the medium for 24 hr. Recombinant TNF-α was then added to the cells, which were cultured for another 72 hr. For further details, see the legend to Fig. 5A.

As discussed above, TNF-α levels were found to increase significantly in culture medium as a result of methylmercury treatment (Fig. 3B). TNF-α that is released extracellularly is known to bind to the TNFR1 present on the cell surface, which then causes cell death^27^,^28^. We next investigated the effect of WP9QY, which inhibits the binding of TNF-α to TNFR1, on the methylmercury sensitivity of C17.2 cells. Upon addition of WP9QY, methylmercury toxicity decreased markedly in a manner dependent on WP9QY concentration (Fig. 5A). In contrast, the single addition of recombinant TNF-α to the culture medium decreased the cell viability (Fig. 5B). From these results, we suggest that the autocrine effect of TNF-α is partially involved in the cellular toxicity caused by methylmercury.

## Discussion

In this study, methylmercury was shown to specifically induce TNF-α expression in brain tissue, and significantly induce TNF-α expression in cultured neural stem cells. TNF-α is an inflammatory cytokine produced by monocytes, macrophages, and fibroblasts. In the central nervous system, astrocytes and microglia are involved in TNF-α production, and in particular, activated microglia produce TNF-α continuously^29^,^30^,^31^,^32^. In addition, TNF-α is known to cause myelin and neuronal damage^33^,^34^. However, it has also been proposed that neuronal damage by TNF-α is not especially pronounced, but rather a result of the production of glutamic acid. Glutamic acid is thought to be a neuronal damaging factor in microglia and is strongly induced by TNF-α, which results in TNF-α indirectly causing the neuronal damage^35^,^36^,^37^. In contrast, methylmercury has been reported to suppress glutamic acid uptake into astrocytes by suppressing Na^+^-dependent transporters^38^. In addition, it has been suggested that astrocytes, in which swelling has been induced by methylmercury, releases glutamic acid extracellularly, which causes neuron death^39^,^40^. Future detailed investigation of glutamic acid production, which is mediated by methylmercury induced TNF-α, may yield important information in explaining the brain-selective expression of methylmercury toxicity.

In addition, although the methylmercury administered in our study to the mice accumulated at a higher concentration in the liver and kidneys than in the brain, the induction of TNF-α expression was found to be most pronounced in brain tissue (Fig. 2). This suggests there is a mechanism in the brain for inducing TNF-α expression that is selectively activated by methylmercury. NF-κB was also shown to participate as a transcription factor in the induction of TNF-α expression by methylmercury (Fig. 4), and so, it is possible that NF-κB may be involved in the selective induction of TNF-α expression in brain tissue. NF-κB is a transcription factor that participates in a variety of bodily responses including the inflammatory response, the immune response, lymphocyte development and differentiation, cell proliferation, and cell death^41^,^42^. In recent years, NF-κB has been reported to participate in the death of neurons from neurodegenerative disorders^43^,^44^. Thus, it appears that NF-κB may play an important role in the central nervous system damage caused by methylmercury. However, NF-κB is also linked to the expression of most cytokines, and has been found to be expressed in many tissues^22^,^23^. In this study, only TNF-α and CD30L, among the 18 members of the TNFSF studied, were shown to induce expression in brain tissue as a result of methylmercury. As such, if NF-κB participates in the induction of TNF-α expression in a brain-tissue-specific manner by methylmercury, it is possible that an unknown induction mechanism mediated by NF-κB may also exist in the brain.

Cytokines are known to regulate the expression of other cytokines, which results in a chain reaction among the cytokines^45^. The interactions of the cytokines involved in such chain reactions, and the cells that produce them, constitute complex cytokine networks^46^,^47^. Recently, we reported that the expression of CCL3 and CCL4, lipopolysaccharide-induced chemokines that are involved in leukocyte infiltration, is selectively induced by methylmercury in the brain^17^. We also recently found that the knockdown of CCL3 or CCL4 conferred the resistance to methylmercury toxicity in C17.2 cells (unpublished data). This result indicates that CCL3 and CCL4, unlike TNF-α, has a protective role against methylmercury toxicity. Because multiple cytokines are induced selectively in the brain by methylmercury, it is also possible that central nervous system damage may be caused by methylmercury as a result of the interaction between these cytokines. In the future, detailed studies of the relationship between the cytokines that are selectively induced in the brain by methylmercury and central nervous system damage caused by methylmercury should shed light on the mechanism behind methylmercury’s toxicity at a molecular level.

## Materials and Methods

### Animal experiments

C57BL/6 male mice (8 weeks old) were purchased from Japan SLC Inc. (Hamamatsu, Japan). Mice were kept in plastic cages (5 animals per cage) under pathogen-free conditions in a room at 22 ± 2 °C and 55 ± 20% relative humidity, with a light/dark cycle of 12 hr. Mice had free access to commercial food that had been sterilized by radiation (CE2; CLEA Japan), and filtered water ad libitum. The Tohoku University Committee for Animal Experiments approved all animal experiments, and the experiments were performed in accordance with the Regulations for Animal Experiments and Related Activities at Tohoku University and Guidelines for Proper Conduct of Animal Experiments by the Ministry of Education, Culture, Sports, Science, and Technology of Japan. After an adaptation period, mice were randomly divided into control (n = 5) and methylmercury-treated groups (n = 5). Methylmercuric chloride, dissolved in physiological saline, was administered via a single subcutaneous injection at a dose of 25 mg/kg. Control mice received injections of saline only. The cerebrums, cerebellums, kidneys, and livers of the mice were dissected under isoflurane anesthesia. Tissue samples were immediately preserved in Isogen II (Nippon Gene, Tokyo, Japan). Aliquots of the samples were taken for analysis of their total mercury content.

### Measurement of TNFSF mRNA expression by real-time quantitative PCR (qPCR)

Total RNA was isolated from mouse tissue and C17.2 cells using an Isogen II kit (Nippon Gene) according to the manufacturer’s protocol. Reverse transcription was performed using 1 μg of total RNA as the template and a PrimeScript^®^ RT reagent kit (Takara, Otsu, Japan) according to the manufacturer’s instructions. qPCR was undertaken using SYBR Premix EX Taq (Takara) and a Thermal Cycler Dice^®^ (Takara) with the oligonucleotides shown in Supplementary Table 1.

### Total mercury determination

The total mercury concentrations in the various tissues of the mice (approximately 10 mg for each tissue) were measured by Zeeman Mercury Spectrometer (RA-915^+^, Lumex Ltd., Russia) based on the atomic absorption spectrometry. This instrument can real-time detect extremely low mercury concentration in biological samples (detection limit was 1 ug/kg). The accuracy of mercury measurement was ensured by using reference NIES CRM No. 13 (National Institute for Environmental Studies, Environmental Agency of Japan).

### Cell culture and treatment

The C17.2 cell line, which is composed of neural stem cells that were derived from the external germinal layer of mouse cerebellum, were cultured in Dulbecco’s modified eagle’s medium supplemented with 10% heat-inactivated fetal bovine serum, 100 IU/mL of penicillin, and 100 mg/mL of streptomycin in a humidified 5% CO_2_ atmosphere at 37 °C. C17.2 cells (4 × 10^5^ cells) were plated in 6-well plates and cultured for 24 hr, followed by treatment with methylmercuric chloride.

### Enzyme-linked immunosorbent assay (ELISA)

TNF-α secreted into culture medium was quantified using a Quantikine ELISA kit (R&D Systems, Minneapolis, MN, USA) according to the manufacturer’s protocol.

### Preparation of nuclear and post-nuclear fractions and immunoblotting

C17.2 cells were washed twice with cold PBS and lysed with a hypotonic buffer (10 mM HEPES-KOH [pH 7.9], 10 mM KCl, 1.5 mM MgCl_2_, 1 mM dithiothreitol [DTT] and 10% Nonidet P-40) containing protease inhibitor (Roche, Indianapolis, IN, USA) for 10 min on ice, and centrifuged at 15,000 × g at 4 °C. The supernatant (post-nuclear fraction) was removed, and the pellet was resuspended in nuclear lysis buffer (10 mM HEPES-KOH [pH 7.9], 400 mM NaCl, 0.2 mM EDTA, 1.5 mM MgCl_2_, and 1 mM DTT, 5% glycerol) containing protease inhibitor (Roche). The resulting suspension was sonicated and centrifuged at 15,000 × g at 4 °C to obtain the nuclear fraction. The protein concentration of each fraction was determined using a DC protein assay kit (Bio-Rad, Hercules, CA, USA). Proteins in nuclear and post-nuclear fractions were separated by SDS-polyacrylamide gel electrophoresis, transferred to an Immobilon-P membrane (Millipore, Bedford, MA, USA), and visualized using primary antibodies against p65 (Santa Cruz Biotechnology, Santa Cruz, CA, USA), lamin A/C (Cell Signaling, Danvers, MA, USA), GAPDH (Santa Cruz Biotechnology), and horseradish peroxidase-coupled secondary antibodies (Dako A/S, Glostrup, Denmark).

## Additional Information

**How to cite this article**: Iwai-Shimada, M. *et al*. Methylmercury induces the expression of TNF-α selectively in the brain of mice. *Sci. Rep.* **6**, 38294; doi: 10.1038/srep38294 (2016).

**Publisher’s note:** Springer Nature remains neutral with regard to jurisdictional claims in published maps and institutional affiliations.

## Supplementary Material

Supplementary Table
