# Supplementary material for: Methylmercury induces the expression of TNF-α selectively in the brain of mice
Source: Sci Rep. 2016 Dec 2;6:38294. doi: 10.1038/srep38294 (PMC5133575; doi:10.1038/srep38294)
Supplement: Supplementary Table [file srep38294-s1.pdf]

Supplementary Table

Methylmercury induces the expression of TNF-α selectively in the brain of mice

Miyuki Iwai-Shimada[a], Tsutomu Takahashi[a, b], Min-Seok Kim[a, c], Masatake Fujimura[c], Hitoyasu Ito[d], Takashi Toyama[a], Akira Naganuma[a] and Gi-Wook Hwang[a, *]

[a] Laboratory of Molecular and Biochemical Toxicology, Graduate School of Pharmaceutical Sciences, Tohoku University, Sendai 980-8578, Japan

[b] School of Pharmacy, Tokyo University of Pharmacy and Life Sciences, 1432-1 Horinouchi, Hachioji, Tokyo 192-0232, Japan

[c] Department of Inhalation Toxicology Research, Korea Institute of Toxicology, Jeonbuk 56212, Republic of Korea

[d] Department of Basic Medical Science, National Institute for Minamata Disease, Kumamoto 867-0008, Japan

[e] Department of Informative Clinical Medicine, Gifu University Graduate School of Medicine, 1-1 Yanagido, Gifu 501-1194, Japan

----

*Address for all correspondence:
Gi-Wook Hwang, Ph.D.
Laboratory of Molecular and Biochemical Toxicology, Graduate School of Pharmaceutical Sciences, Tohoku University, Sendai 980-8578, Japan
Phone & Fax: +81-22-795-6872
E-mail: gwhwang@m.tohoku.ac.jp

Supplementary Table 1. Oligonucleotide primers used for real-time quantitative PCR

Gene	Sense (5′→3′)	Antisense (5′→3′)
TNF-ß (TNFSF1)	GACTCTCTGGTGTCCGCTTC	AGAGAAGCCATGTCGGAGGA
TNF-α (TNFSF2)	CGTCAGCCGATTTGCTATCT	CGGACTCCGCAAAGTCTAAG
LT-ß (TNFSF3)	TATCACTGTGCCTGGCTGTGC	TCCTGGAAGCATTGGATCTC
TNFSF4	CCCTCCAATCCAAAGACTCA	ATCCTTCGACCATCGTTCAG
CD40L (TNFSF5)	AAAATGGGAAACAGCTGACG	GGTATTTGCCGCCTTGAGTA
FasL (TNFSF6)	CATCACAACCACTCCCACTG	GTTCTGCCAGTTCCTTCTGC
TNFSF7	AGGCTGCATATCCAGGTGAC	CAGGTATGTCAGGCGCTGTA
CD30L (TNFSF8)	GGCCTACCTCCAAGTGTCAA	TGATGAGGAGCTGCAATGTC
TNFSF9	ATTCACAAACACAGGCCACA	GATAAGCCCTCAGACCCACA
TNFSF10	CCCTGCTTGCAGGTTAAGAG	GGCCTAAGGTCTTTCCATCC
RANKL (TNFSF11)	TATACTTTCGAGCGCAGATG	CCACAATGTGTTGCAGTTCC
TNFSF12	AGGAGGAGCTGACAGCAGAG	GCCGAGGATGAACCTCATAA
TNFSF13	TCAGGTGGTATCTCGGGAAG	AATGTTCCATGCGGAGAAAG
TNFSF13B	TTCCATGGCTTCTCAGCTTT	CGTCCCCAAAGACGTGTACT
TNFSF13C	GACCCTGGTGGGTCTAGTGA	GTAGGAGCTGAGGCATGAGG
TNFSF14	GTGTTTGTGGTGGATGGACA	TCTCCAAGACGTTGATGCAG
TNFSF15	TCCCCGGAAAAGACTGTATG	ACTTCATCCCGTTCTTGGTG
TNFSF18	CAAGTCCTCAAAGGGCAGAG	AGCTTCCCATCAGATGTCGT
